# Association Between Playing Surface and Anterior Cruciate Ligament (ACL) Injury Risk in the National Football League: A Systematic Review and Exploratory Meta-Analysis

**DOI:** 10.7759/cureus.113994

**Published:** 2026-08-05

**Authors:** Vivek Vemugunta, Aadil Touheed, Arjun Mahamkali, Hayden Runnells, Hritik Nerusu, Lisa Cannada

**Affiliations:** 1 Orthopedics, University of Central Florida College of Medicine, Orlando, USA; 2 Orthopedics, New York Institute of Technology College of Medicine, New York City, USA; 3 Sports Medicine, University of Central Florida College of Medicine, Orlando, USA; 4 Orthopedic Surgery, Novant Health, Charlotte, USA

**Keywords:** acl, anterior cruciate ligament, artificial turf, meta-analysis, national football league, natural grass, playing surface, sports injury epidemiology, synthetic turf, systematic review

## Abstract

In professional sports, anterior cruciate ligament (ACL) ruptures are among the most consequential injuries, especially in American football. This review set out to determine whether National Football League (NFL) players are more likely to sustain ACL injuries on artificial turf than on natural grass. To answer this, studies were gathered from PubMed, and the review process followed PRISMA 2020 guidelines to ensure clear and reliable reporting. Ten studies met the inclusion criteria: eight on NFL epidemiology or clinical outcomes, one systematic review covering multiple sports, and one biomechanical study. For all studies with multiple populations, data extraction and analysis were limited to the NFL stratum, comparing ACL injury outcomes between artificial turf and natural grass within this group of professional athletes. When looking at the total number of ACL injuries across NFL studies, the counts were either close between artificial turf and natural grass or slightly higher on grass. But after adjusting for how often each surface was used, the rate of ACL injuries was the same or higher on artificial turf. This pattern held whether exposure was expressed per game or per team-game, and whether the comparison was reported as an injury rate, an incidence-density ratio, or an odds ratio; no individual comparison reached statistical significance. Through an exploratory random-effects meta-analysis of four recent NFL studies, calculated through the Hartung-Knapp method, we found a small increase in injury rates on turf, with a pooled estimate modestly above the null. This trend remained steady even when each study was left out one by one. However, the difference wasn't statistically significant, and because these studies often overlapped in the seasons they covered, the authors view these results as an early indication rather than a definitive conclusion. Other findings back up this tendency: knee injuries overall, and noncontact knee injuries in particular, occurred at higher rates on synthetic turf, and season-ending surgeries, usually ACL reconstructions, were also more common after injuries sustained on turf. Previous pooled studies show that the increased risk of ACL injury is specific to American football, while biomechanical evidence suggests that natural grass reduces the force transmitted through the cleat. None of the NFL studies show a statistically significant increase in ACL injuries on artificial turf when considered individually. However, after accounting for exposure, the direction of the data, results from pooled analyses, higher rates of season-ending surgery, and biomechanical findings all suggest that ACL injuries could be more common on synthetic surfaces. A clearer understanding of these trends will be essential for guiding future decisions on field surfaces and supporting the long-term health and safety of NFL athletes.

## Introduction and background

Anterior cruciate ligament (ACL) injuries represent a substantial clinical concern within all professional sports, but especially within American football, as they make up approximately 2% of all reported injuries in the NFL. An ACL tear for any National Football League (NFL) player is typically a season-ending event: surgical reconstruction and an extended period of rehabilitation are almost always required. Even after successful reconstruction, a substantial proportion of players do not return to their pre-injury level of performance, and ongoing surveillance aims to clarify the frequency, mechanisms, and modifiable risk factors of these injuries [1,2].

The type of playing surface is a central aspect in discussions about ACL injury risk. In recent years, modern infill synthetic turf has become more common in NFL stadiums, used alongside natural grass. Laboratory tests show that natural grass tends to release the cleat by tearing or forming a divot, whereas synthetic turf holds the cleat more firmly and transmits more horizontal force and rotational torque to the leg during movement [3]. Because a large share of ACL injuries in football occur during noncontact actions such as deceleration, cutting, or pivoting, differences in how surfaces interact with footwear may help explain why artificial turf could be linked to a higher risk of ACL and other lower-limb injuries [3,4].

It remains challenging to identify a clear clinical signal for this mechanism. Across the league, broader analyses have found higher rates of lower-extremity injuries occurring on synthetic turf [4]. However, when focusing on ACL injuries, the findings are less consistent. Some NFL studies have found similar numbers of ACL injuries on both surfaces, and in some cases, more injuries have occurred on natural grass. A previous systematic review that examined both football and soccer found that the increased ACL risk on synthetic surfaces was limited to American football, with no similar effect observed in soccer. This highlights how sport-specific factors may shape the relationship between surface type and injury risk [5].

Making sense of these results is further complicated by using raw injury counts without adjusting for how much each surface is used, differences in how outcomes are defined (e.g., ACL-specific versus all lower-extremity injuries), and changes in playing surfaces over time.

Despite ongoing debate about whether playing surface affects injury risk, no review has singled out ACL injuries in the NFL until now. Past studies have either grouped football with other sports, such as soccer, or examined a wide range of athletic settings. This review, on the other hand, zeroes in on the NFL alone. Instead of just adding up the number of injuries, this review looks at how frequently ACL injuries happen in relation to the actual time players spend playing. The analysis uses recent information, including data from the 2024-2025 season. A first round of statistical analysis is included as well. By taking these steps, the review is able to offer a timely and thorough look at the issue. The main goal of this review is to determine whether playing on artificial turf increases NFL athletes' risk of ACL injuries compared with natural grass, whether during games or practices. This investigation was organized using the Population, Exposure, Comparator, Outcome (PECO) framework rather than the Population, Intervention, Comparison, and Outcome (PICO) framework because playing surface is an exposure rather than an intervention. Here, NFL players make up the population, artificial turf is considered the exposure, natural grass is the comparison, and ACL injury is the outcome being evaluated.

Materials and methods

Protocol and Registration

This systematic review was not registered in a public registry, and a formal review protocol was not prepared in advance. The review was nonetheless conducted and reported in accordance with the PRISMA 2020 statement [6].

Eligibility Criteria

Studies were eligible if they addressed the review question in an NFL or professional American population. The full inclusion and exclusion criteria, organized by PECO element, are presented in Table 1. In brief, eligible reports compared artificial turf with natural grass and reported an ACL outcome, or a directly relevant surface-specific lower-extremity or biomechanical outcome; one prior systematic review and one biomechanical study were retained to provide a pooled and mechanistic context for the NFL-specific findings.

**Table 1:** Eligibility criteria for study inclusion, organized by PECO element ACL: anterior cruciate ligament; NCAA: National Collegiate Athletic Association; NFL: National Football League; PECO: Population, Exposure, Comparator, Outcome

PECO element	Inclusion criteria	Exclusion criteria
Population	NFL players during games and/or practices; professional American-football populations and surfaces where used for mechanistic or pooled context	Collegiate (NCAA), high-school, recreational, or non-American-football populations as the sole study group
Exposure	Artificial (synthetic/infill) turf, including hybrid surfaces	Studies without an identifiable artificial-surface exposure
Comparator	Natural grass playing surface	Absence of a turf-versus-grass comparison
Outcome	ACL injury (counts, rates, or risk estimates); surface-specific lower-extremity injury or cleat–surface biomechanics accepted as supporting outcomes	Outcomes unrelated to ACL or to surface-specific lower-extremity injury
Study type	Primary epidemiologic, clinical, or biomechanical studies; one prior systematic review retained for pooled context	Narrative reviews, editorials, commentaries, and conference abstracts
Other	English language; full text retrievable	Duplicate or overlapping data without extractable surface-stratified results

Information Sources and Search Strategy

PubMed/Medical Literature Analysis and Retrieval System Online (MEDLINE) served as the information source. The search combined controlled vocabulary and free-text terms for the ACL, the NFL, and playing surface, of the general form: (“Anterior Cruciate Ligament Injuries”[MeSH] OR “anterior cruciate ligament”[tiab] OR ACL[tiab]) AND (“National Football League”[tiab] OR NFL[tiab]) AND (“artificial turf”[tiab] OR “synthetic turf”[tiab] OR “natural grass”[tiab] OR “playing surface”[tiab] OR “field surface”[tiab] OR FieldTurf[tiab]).

Study Selection

The PubMed/MEDLINE search was executed in October 2025. Screening followed a two-step process: titles and abstracts were screened first, then full texts were assessed against the eligibility criteria. Screening was carried out by the review team, with any disagreements resolved by consensus.

Data Extraction

Data extraction was structured around three categories: (1) study and population details (author, year, design, level of evidence, season, sample size, data source); (2) exposure and playing surface information (surface type and, where available, exposure metrics for example as games, team-games, plays, or athlete-exposures); and (3) outcome data (ACL injury counts and rates by surface, effect estimates with confidence intervals, and findings by exposure setting or injury mechanism). For studies that included multiple athlete groups, only data from NFL athletes were analyzed.

Five reviewers handled the data extraction, with each assigned a portion of the studies. Once extraction was complete, another team member compared the collected data to the original set of sources and corrected any errors as needed. If any differences came up, the team met to resolve them together. Contact with study authors was not made. Effect estimates were taken as reported, except during the exploratory meta-analysis, where the team calculated rate ratios and standard errors using the ACL counts and surface-specific game denominators. When both raw counts and exposure-adjusted rates were available for a comparison, the exposure-adjusted value was prioritized. All studies, including those with overlapping data, were included, and any influence of this data overlap on the pooled results is discussed in the Data Synthesis and Limitations sections.

Risk of Bias and Level of Evidence

Risk of bias was assessed with Risk Of Bias In Non-randomised Studies of Exposures (ROBINS-E), a domain-based instrument developed for observational studies of exposure effects and suited to the PECO structure of this review [7]. ROBINS-E was applied to eight NFL observational studies; only those that reported a surface-specific exposure-outcome comparison were included. For each study, the result assessed was the comparison of ACL injury frequency or rate between artificial turf and natural grass; for the two studies that report no ACL-specific surface rate, the closest surface-specific knee or season-ending-surgery outcome was assessed. Studies were rated across the seven ROBINS-E domains and assigned domain-level and overall judgements of low risk of bias, some concerns, high risk of bias, or very high risk of bias. Domain-level and overall judgements were derived from the source articles and reviewed by the review team, with any disagreement resolved by consensus. Confounders specified in advance as important were surface-specific exposure, exposure setting (game versus practice), weather and surface temperature, surface generation, and player position. No numerical quality score was derived, as ROBINS-E is based on domain-level judgements rather than summary scores.

ROBINS-E does not apply to the two non-epidemiological studies. The included systematic review [5] was appraised with A MeaSurement Tool to Assess Systematic Reviews 2 (AMSTAR 2) [8], and the biomechanical study [3], for which no validated instrument exists, was appraised narratively as neither contributes NFL incidence data.

Data Synthesis

A descriptive approach was used to synthesize the evidence, as the included studies differed widely in both clinical and methodological aspects. For example, some studies reported exposure by games, others by team games, plays, or athlete exposures. Definitions of outcomes also varied, ranging from ACL-specific injuries to all lower-extremity injuries, and the types of playing surfaces analyzed covered several generations from 1986 to 2023. Notably, a previous systematic review on this topic also found that pooling all studies together was not practical due to these differences [5]. To make this more systematic, the results were organized into several main areas: how ACL injuries are evaluated across playing surfaces; the influence of different exposure settings and injury mechanisms; broader trends in lower-extremity injuries and surgeries; and what pooled or biomechanical studies show. When the data permitted, rates were adjusted for exposure rather than simply reporting the number of injuries, to make comparisons clearer.

Statistical Analysis

An exploratory meta-analysis was restricted to the four NFL studies [2,9-11] reporting ACL injury counts with a surface-specific game denominator (games or team-games for Dodson et al. [2]). In the analysis by McCormick et al. [11], only the specific numbers for ACL injuries were used, with 81 cases on artificial turf and 50 on natural grass. Rather than including all knee ligament injuries, the focus remained on ACL events alone. For Card et al. [10], the main analysis grouped artificial turf and hybrid GrassMaster surfaces together, while a separate sensitivity analysis examined artificial turf on its own. Each study's log rate ratio was pooled using a random-effects model that incorporated the Hartung-Knapp adjustment [12], as this approach is considered more dependable for analyses involving few studies. Consistency between studies was evaluated with the I² statistic and Cochran's Q test, though their accuracy is limited when the dataset is this small. To check the stability of the results, the analysis was repeated, leaving out one study at a time. A funnel plot was generated to visually check for small-study effects; formal tests such as Egger's test were not used, since they are not reliable for fewer than 10 studies. Because some of the studies covered overlapping periods and used different denominators, the pooled results are presented as preliminary observations, not as final conclusions. All statistical analyses were completed using Python 3 (Python Software Foundation, Wilmington, DE, USA) [13].

## Review

Results

Study Selection

**Figure 1:**
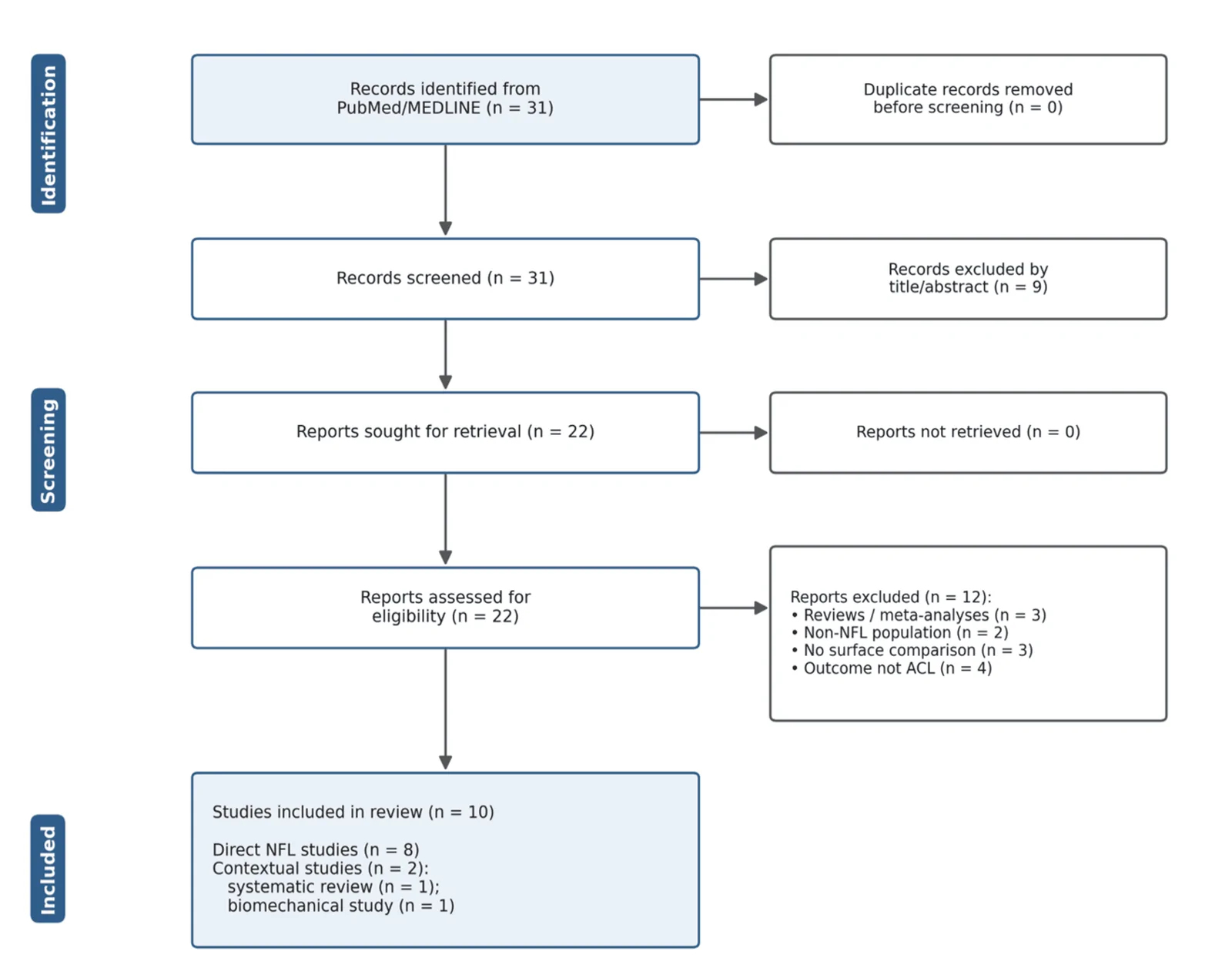
PRISMA 2020 flow diagram of study identification, screening, eligibility, and inclusion. A single database (PubMed/MEDLINE) was searched, and records were screened in two sequential stages, first by title and abstract and then by full text, against the a priori PECO eligibility criteria, with disagreements resolved by consensus. Of 31 records identified, 9 were excluded at title/abstract screening; of the 22 full-text articles assessed, 12 were excluded (reviews or meta-analyses, n = 3; non-NFL populations, n = 2; no surface comparison, n = 3; outcome not ACL, n = 4), leaving 10 studies for qualitative synthesis. ACL: anterior cruciate ligament; NFL: National Football League; PECO: Population, Exposure, Comparator, Outcome; MEDLINE: Medical Literature Analysis and Retrieval System Online

The PubMed search identified 31 records, with no duplicates removed before screening. Nine records were excluded at the title/abstract stage, leaving 22 reports that were sought and assessed in full. Twelve reports were subsequently excluded for the following reasons: reviews and meta-analyses (n = 3), non-NFL populations (n = 2), studies without a surface comparison (n = 3), and studies whose outcomes were not ACL injuries (n = 4). Ten studies met all criteria and were included in the synthesis (Figure 1).

Characteristics of the Included Studies

The 10 included studies were published between 1997 and 2025 (Table 2). Eight were NFL-specific epidemiologic or clinical studies, most of which were Level III retrospective cohorts that drew on league or public injury records and spanned 1986 through 2023. The remaining two were a prior systematic review of synthetic surfaces and ACL injury across football and soccer [5] and a biomechanical study of the cleat-surface interface for surfaces used by professional American teams [3]; both were retained as contextual evidence, cross-sport and mechanistic, respectively, and were not treated as direct NFL ACL incidence studies or combined with the NFL cohorts in the meta-analysis.

**Table 2:** Characteristics of the 10 included studies ACL: anterior cruciate ligament; ISS: Injury Surveillance System; LE: lower extremity; NFL: National Football League; EMBASE: Excerpta Medica Database; PROSPERO: International Prospective Register of Systematic Reviews

Study	Design; level of evidence	Population & seasons	Sample size	Primary data source
Garlapaty et al., 2025 [9]	Retrospective cohort, descriptive epidemiology; Level III	NFL players, all positions; 2012–2022 (11 seasons)	520 ACL tears	Public databases, game film, team injury reports
Card et al., 2024 [10]	Retrospective epidemiologic cohort; Level III	NFL athletes; 2017–2021 (pre-, regular season, playoffs)	173 ACL injuries with known surface	Publicly available injury and game data
McCormick et al., 2024 [11]	Retrospective cohort; Level III	NFL in-game knee-ligament tears; 2020– 2023 (incl. preseason)	149 knee-ligament injuries	Publicly available injury and game data
Dodson et al., 2016 [2]	Descriptive epidemiology; Level III	NFL athletes; 2010– 2013	219 ACL injuries	Public news reports; USA Today game database
Bradley et al., 2002 [1]	Descriptive (NFL ISS) + team-physician survey	NFL players; NFL ISS (1986–1995; detailed 1994–1998)	209 ACL injuries; survey of 31 team physicians	NFL Injury Surveillance System
Venishetty et al., 2024 [14]	Descriptive epidemiology; Level III	NFL lower-extremity injuries; 2021–2022	718 lower-extremity injuries	Publicly available injury data
Mack et al., 2019 [4]	Cohort; Level III	NFL athletes, all 32 teams; 2012–2016 regular-season games	4,801 LE injuries; 1,280 games	Mandated league-wide injury surveillance
Balazs et al., 2015 [5]	Systematic review	Football & soccer cohorts (including NFL); prospectively gathered data	10 studies; 963 ACL injuries	OVID, EMBASE, Cochrane, PROSPERO
Scranton et al., 1997 [15]	Comparative case series; Level IV	22 NFL teams; 1989– 1993	61 noncontact ACL injuries (of 78 reported)	NFL Injury Surveillance System
Kent et al., 2015 [3]	Biomechanical (mechanistic) study; Level V	Eight surfaces used by professional American-football teams (2 grass, 6 infill)	Cleat–surface mechanical testing	Laboratory testing apparatus

ACL Injury by Playing Surface

Table 3 shows the distribution of ACL injuries by playing surface. In the largest study, Garlapaty and colleagues looked at 520 ACL tears across 11 NFL seasons. They found that injuries occurred with almost the same frequency on artificial turf (265 cases) and natural grass (255 cases). The per-game rates were 0.061 for turf and 0.058 for grass, with no statistically significant difference between the two surfaces, whether measured by injury counts (p = 0.670) or by rates adjusted for exposure (p = 0.661) [9]. Dodson et al. recorded more in-game ACL injuries on grass (74) than on turf (63); yet, after accounting for the greater number of teams played on grass, the per-team-game rate was marginally higher on turf (0.053 vs. 0.050; p > .05) [2]. Card et al. observed 100 ACL injuries on non-natural surfaces (85 turf plus 15 GrassMaster) versus 73 on grass; per-game rates were 0.115 on turf, 0.134 on GrassMaster, and 0.097 on grass, and the odds of ACL injury on non-natural relative to grass surfaces were not significantly elevated (odds ratio 1.239; 95% CI 0.900-1.704; p = 0.189) [10].

**Table 3:** ACL injury by playing surface across the included studies Counts are raw injury numbers unless a rate is given; rates are not adjusted for surface-specific exposure beyond what each study reported. *Card et al. [10] non-natural total comprises 85 turf + 15 GrassMaster. †McCormick et al. ACL tears across 2020–2023 (turf 17/26/22/16; grass 13/16/10/11); rates shown are ACL-specific (81/689 and 50/524 per game); the study's significance test (p = 0.379) was performed on all knee-ligament injuries (turf 91, grass 58; 0.132 vs 0.111 per game). Surface-specific ACL counts not applicable (lower-extremity, systematic-review, or biomechanical study). ACL: anterior cruciate ligament; CI: confidence interval; IDR: incidence-density ratio; IRR: incidence rate ratio; NS: not significant; LE: lower extremity; OR: odds ratio; RR: rate ratio; recon: reconstruction

Study	Artificial turf	Natural grass	Surface comparison (effect; significance)
Garlapaty et al., 2025 [9]	265 (0.061/game)	255 (0.058/game)	No significant difference; counts p = 0.670, game incidence p = 0.661
Dodson et al., 2016 [2]	63 (0.053/team-game)	74 (0.050/team-game)	Rate marginally higher on turf; P > .05 (NS)
Card et al., 2024 [10]	100* (0.118/game)	73 (0.097/game)	OR 1.239 (95% CI 0.900–1.704), p = 0.189; rate ratio 1.21 (NS)
McCormick et al., 2024 [11]	ACL 81† (0.118/game)	ACL 50† (0.095/game)	More ACL on turf; knee-ligament rate NS (p = 0.379)
Scranton et al., 1997 [15]	21 noncontact	40 noncontact	Overall incidence-density ratio 1.59 (higher rate on artificial)
Bradley et al., 2002 [1]	Game 71; practice 12	Game 71; practice 55	Equal in games; practice excess on grass attributed to exposure
Mack et al., 2019 [4]	—	—	Knee IRR 1.20 (1.07–1.34); noncontact knee 1.46 (1.20–1.77)
Venishetty et al., 2024 [14]	ACL recon 30	ACL recon 17	Season-ending-surgery OR 1.60 (1.28–1.99); LE 1.42 vs 1.22/game
Balazs et al., 2015 [5]	—	—	4 football cohorts ↑ on synthetic (RR 1.1–1.92; 3 significant); soccer none
Kent et al., 2015 [3]	—	—	Greater force/torque on artificial; natural grass force-limits via tearing

McCormick et al. examined 149 in-game knee-ligament injuries from 2020 to 2023; the ACL was the most commonly injured ligament every year, and ACL tears were more frequent on artificial turf than on natural grass (81 vs 50 overall: 17 vs 13 in 2020, 26 vs 16 in 2021, 22 vs 10 in 2022, and 16 vs 11 in 2023), although the overall in-game knee-ligament rate did not vary substantially between surfaces (0.132 vs 0.111 per game; combined p = 0.379) [11]. In the earliest comparative series, Scranton et al. identified 61 surgically confirmed noncontact ACL injuries across 22 NFL teams (1989-1993); although raw counts were higher on natural grass (40, 65.6%) than on artificial surfaces (21, 34.4%), the exposure-adjusted overall incidence-density ratio was 1.59, showing a higher noncontact ACL rate on artificial surfaces [15]. Bradley et al., reviewing NFL Injury Surveillance System data, reported 209 ACL injuries; in games, the number was equal on both surfaces (71 each), whereas the practice excess on grass (55 vs. 12) was attributed to the fact that early-season practices are conducted almost entirely on natural grass [1].

Influence of Exposure Setting and Injury Mechanism

Several studies stratified the surface effect by exposure setting and mechanism (Table 4). Garlapaty et al. found that 67.7% of ACL tears occurred in games and that injury mechanism did not differ by surface (contact 58.8% vs. noncontact 41.2%; p = 0.702) [9]. Scranton and colleagues found that the impact of the playing surface varied by setting. In games, the rate of noncontact ACL injuries was higher on grass, with an incidence-density ratio of 0.19. In contrast, during practices, noncontact ACL injuries occurred much more frequently on artificial turf, with an incidence-density ratio of around 4.0. When combining both settings, the overall ratio was 1.59, showing a higher risk associated with artificial surfaces [15]. Bradley et al. similarly found game ACL injuries to be equal across surfaces, with the apparent grass predominance confined to practices and explained by exposure [1]. Mack et al. found that the association involving synthetic turf and injuries became stronger when injuries were restricted to noncontact or surface-contact mechanisms, which are the categories most plausibly linked to shoe-surface release; the knee incidence rate ratio in this subgroup was 1.46 (95% CI 1.20-1.77). Injuries attributed to player contact, by contrast, showed no difference between surfaces [4].

**Table 4:** Surface effect by exposure setting and injury mechanism in the NFL-specific studies NFL: National Football League; ACL: anterior cruciate ligament; IDR: incidence-density ratio; IRR: incidence rate ratio

Study	Exposure setting	Injury mechanism	Net NFL surface signal
Garlapaty et al., 2025 [9]	67.7% in games; game incidence 0.061 turf vs 0.058 grass (p = 0.661)	Contact 58.8% / noncontact 41.2%; no surface difference (p = 0.702)	No significant difference
Scranton et al., 1997 [15]	Game rate higher on grass (IDR 0.19); practice rate higher on artificial (IDR ≈ 4.0)	Noncontact ACL only	Overall rate higher on artificial (IDR 1.59)
Bradley et al., 2002 [1]	Game ACL equal by surface (71/71); practice excess on grass (55 vs 12)	Mixed (contact, blocking, noncontact)	Game-equal; practice excess driven by exposure
Mack et al., 2019 [4]	Regular-season games (2012– 2016)	Noncontact/surface-contact knee IRR 1.46 (1.20–1.77); player-contact: no difference	Knee injury higher on synthetic

Broader Lower-Extremity and Surgical Outcomes

Two studies evaluated surface effects beyond the ACL. Mack et al. analyzed mandated league-wide surveillance from 2012 to 2016 (1,280 games; 4,801 lower-extremity injuries) and found a 16% higher lower-extremity injury rate per play on synthetic turf (incidence rate ratio 1.16; 95% CI 1.10-1.23); the knee subgroup, which anatomically includes the ACL, was also significantly elevated (any-time-loss incidence rate ratio 1.20; 95% CI 1.07-1.34) [4]. Venishetty et al. reported a higher lower-extremity injury rate on turf than on grass (1.42 vs 1.22 per game) for 2021-2022 and significantly greater odds of season-ending surgery on artificial turf (odds ratio 1.60; 95% CI 1.28-1.99); ACL reconstruction was the most common season-ending procedure, performed after 30 turf injuries versus 17 grass injuries [14].

Exploratory Meta-Analysis of ACL Incidence by Surface

**Figure 2:**
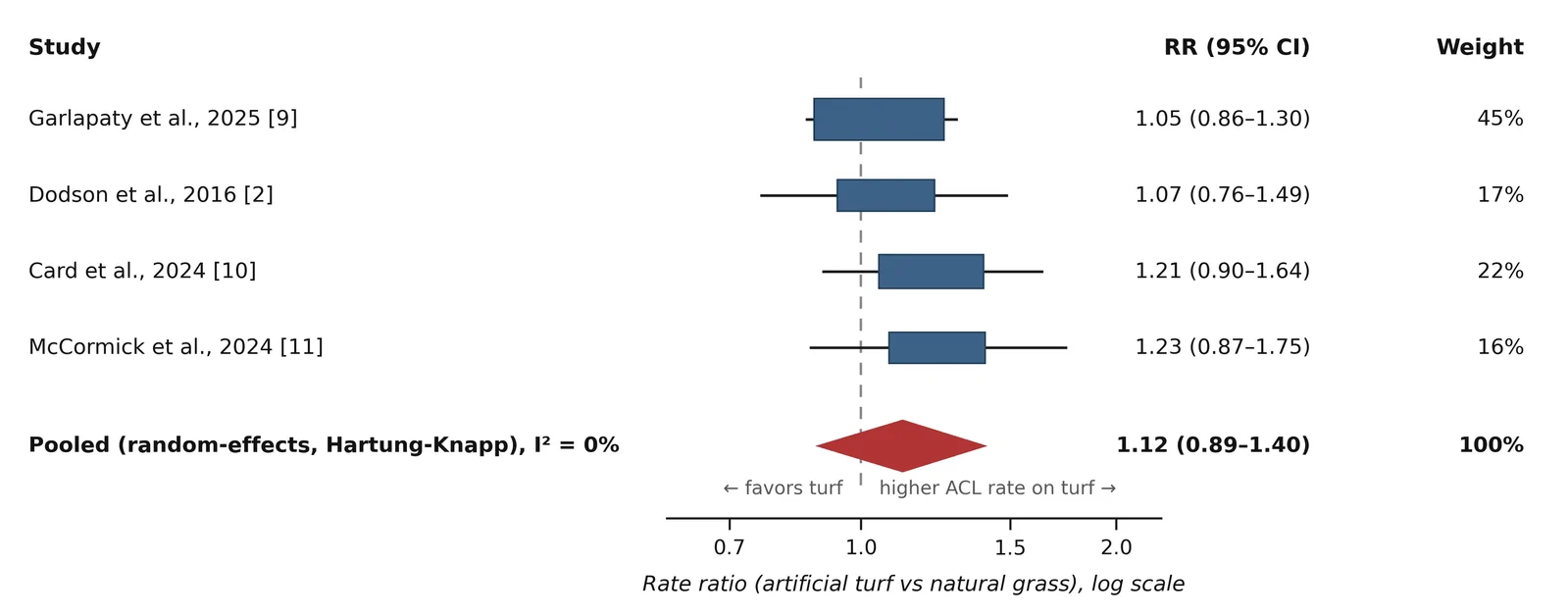
Exploratory random-effects meta-analysis of ACL incidence by playing surface in modern NFL game-level cohorts. The four NFL studies reporting ACL injury counts with a surface-specific game denominator were included (Garlapaty et al. [9], Dodson et al. [2], Card et al. [10], and McCormick et al. [11]). For each study, a rate ratio comparing artificial turf with natural grass was calculated, with the standard error derived from the surface-specific ACL counts under a Poisson assumption; for McCormick et al. [11], the ACL-specific counts were used, and for Card et al. [10], the study's non-natural (turf plus hybrid) grouping was retained. Log rate ratios were pooled with a random-effects model using the Hartung-Knapp adjustment (I² = 0%; Cochran Q = 0.93, df = 3, p = 0.82). Squares represent study-specific rate ratios, sized by inverse-variance weight; horizontal lines are 95% confidence intervals; the diamond is the pooled estimate (rate ratio 1.12, 95% CI 0.89-1.40); the dashed line marks the null (rate ratio = 1.0). Analyses were performed in Python 3 (Python Software Foundation, Wilmington, DE) [13]. ACL: anterior cruciate ligament; CI: confidence interval; NFL: National Football League; RR: rate ratio.

**Figure 3:**
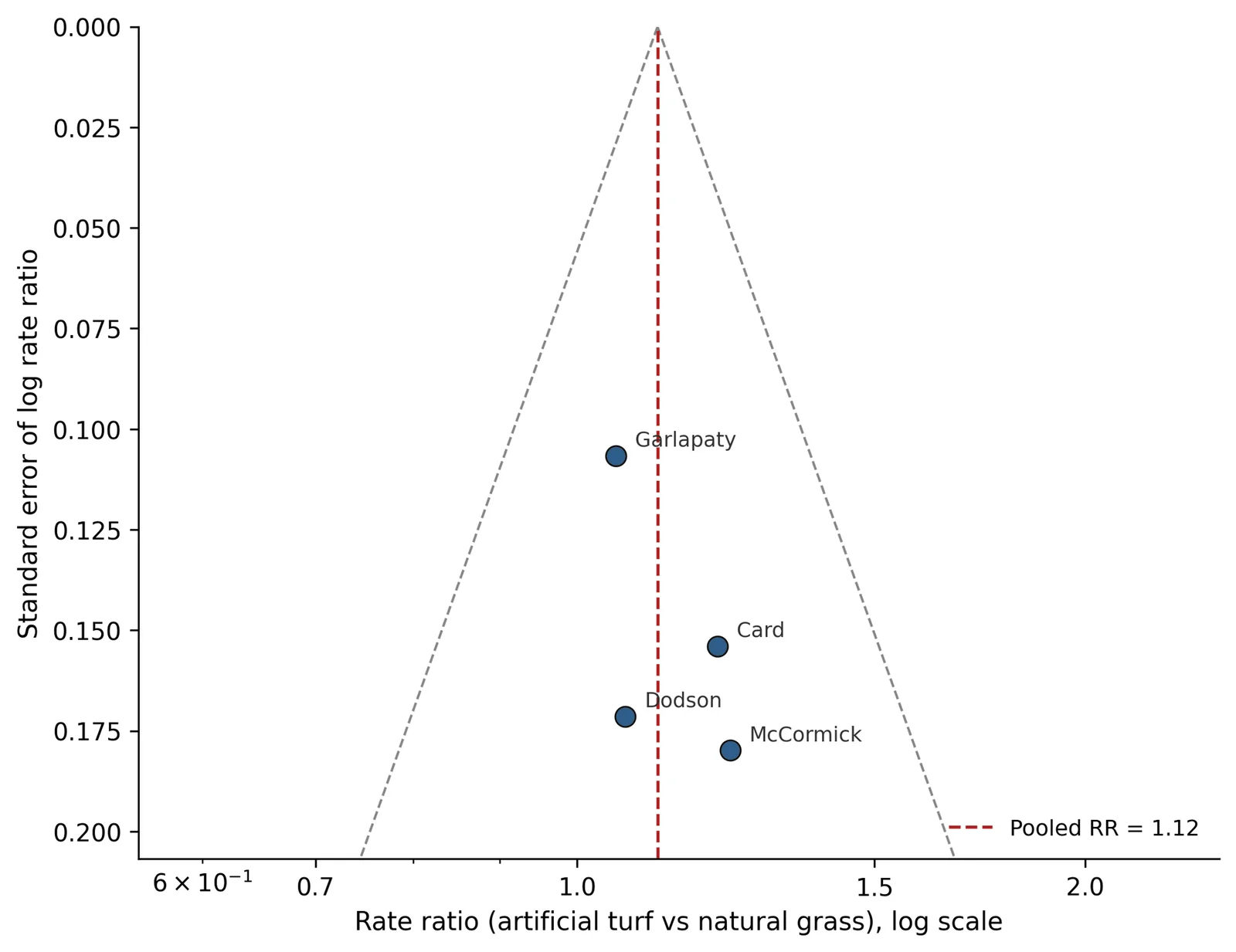
Funnel plot of the four NFL cohorts in the exploratory meta-analysis of ACL incidence by playing surface. The vertical dashed line marks the pooled rate ratio (1.12) and the diagonal dashed lines the pseudo-95% confidence region. All four studies fall within this region, with the more precise studies nearest the pooled estimate. With only four studies, funnel-plot asymmetry cannot be assessed formally, and no statistical test (e.g., Egger's) was performed. All analyses, including this funnel plot, were performed in Python 3 (Python Software Foundation, Wilmington, DE, USA) [13]. NFL: National Football League; ACL: anterior cruciate ligament; RR: rate ratio.

Four NFL studies reported ACL injury counts with a surface-specific game denominator and were eligible for pooling (Dodson et al. [2], Garlapaty et al. [9], Card et al. [10], and McCormick et al. [11]; Table 5). Each study's rate ratio favored a higher ACL rate on artificial turf, ranging from 1.05 to 1.23, and none was individually significant. After combining the data using a Hartung-Knapp random-effects model, the rate ratio comparing surfaces was 1.12, with a 95% Cl of 0.89 to 1.40 (Figure 2). Because the confidence interval includes one, this result does not show a statistically significant difference between artificial turf and natural grass. However, the trend observed suggests a higher rate of ACL injuries on artificial turf. The result was stable in a leave-one-out analysis, in which the pooled rate ratio remained between 1.09 and 1.17 regardless of which study was removed, and in a sensitivity analysis restricting Card et al. to the artificial-turf stratum alone (excluding the hybrid GrassMaster surface; rate ratio 1.11, 95% CI 0.88-1.40) [10]. The Cochran Q test did not show statistical significance (Q = 0.93, df = 3, p = 0.82), and the I² statistic was calculated as 0%. Even so, because only four cohorts had overlapping time periods, these measures are not very precise. The I² value, in particular, should not be taken as clear evidence that the studies are truly consistent with one another. Because the cohorts covered similar years, a single ACL injury could potentially appear in more than one dataset. For this reason, the combined result should be viewed as exploratory rather than a definitive finding. A funnel plot of the four pooled studies is shown in Figure 3. All four studies fall within the pseudo-95% confidence region, with the more precise studies lying closest to the pooled estimate and the two smaller studies showing somewhat higher rate ratios; however, with only four cohorts, this pattern is as likely to reflect chance as any systematic small-study effect. Formal tests of funnel-plot asymmetry, such as Egger's test, were not performed, as they are unreliable when fewer than 10 studies are available.

**Table 5:** Meta-analysis of ACL injuries by playing surface in recent NFL game-level cohorts For each study, the number of ACL injuries on a given surface was divided by the number of games (or team-games, as in Dodson et al.) played on that surface. Rate ratios compare the risk of ACL injury on artificial turf to that on natural grass. In the study by Card and colleagues, the analysis evaluated non-natural surfaces (artificial turf plus hybrid fields) with natural grass. The pooled estimate used the Hartung-Knapp random-effects method; for McCormick et al., ACL-specific counts are used; a sensitivity analysis restricting Card et al. to artificial turf alone gave 1.11 (95% CI 0.88–1.40). NFL: National Football League; CI: confidence interval; RR: rate ratio.

Study	Turf events/exposure	Grass events/exposure	Rate ratio (95% CI)	Weight
Garlapaty et al., 2025 [9]	181/2,980 games	171/2,968 games	1.05 (0.86–1.30)	45%
Dodson et al., 2016 [2]	63/1,178 team-games	74/1,478 team-games	1.07 (0.76–1.49)	17%
Card et al., 2024 [10]	100/851 games	73/752 games	1.21 (0.90–1.64)	22%
McCormick et al., 2024 [11]	81/689 games	50/524 games	1.23 (0.87–1.75)	16%
Pooled (random-effects, Hartung-Knapp), I² = 0%	—	—	1.12 (0.89–1.4)	100%

Table 5 summarizes the exploratory meta-analysis of ACL injuries by playing surface in recent NFL game-level cohorts. For each study, the number of ACL injuries on a given surface was divided by the number of games (or team-games, as in Dodson et al. [2]) played on that surface. Rate ratios compare the risk of ACL injury on artificial turf to that on natural grass. In the study by Card and colleagues [10], the analysis evaluated non-natural surfaces (artificial turf plus hybrid fields) with natural grass. 

Pooled and Biomechanical Evidence

Balazs et al. systematically reviewed 10 studies (963 ACL injuries) across football and soccer and found that the four American-football cohorts demonstrating an increased risk of ACL injury on artificial surfaces did so across both early-generation and modern infill turf (rate ratios approximately 1.1 to 1.92; three statistically significant), whereas no soccer cohort showed such an increase; like the present review, that study could not pool all of its data owing to incompatible exposure units [5]. On a mechanical level, Kent and colleagues studied how football cleats interacted with eight different surfaces used in professional leagues. Their findings showed that natural grass helped limit horizontal force, as the surface tore under pressure. The study also found that artificial turf generated higher horizontal forces, measuring 4.5 kN compared to 3.2 kN for natural grass. The study also examined rotational torque and found it was higher on turf (197 N·m) than on natural grass (145 N·m). This difference reached statistical significance. These results suggest that natural grass is better at absorbing and distributing force during play than synthetic turf [3].

Risk of Bias

**Figure 4:**
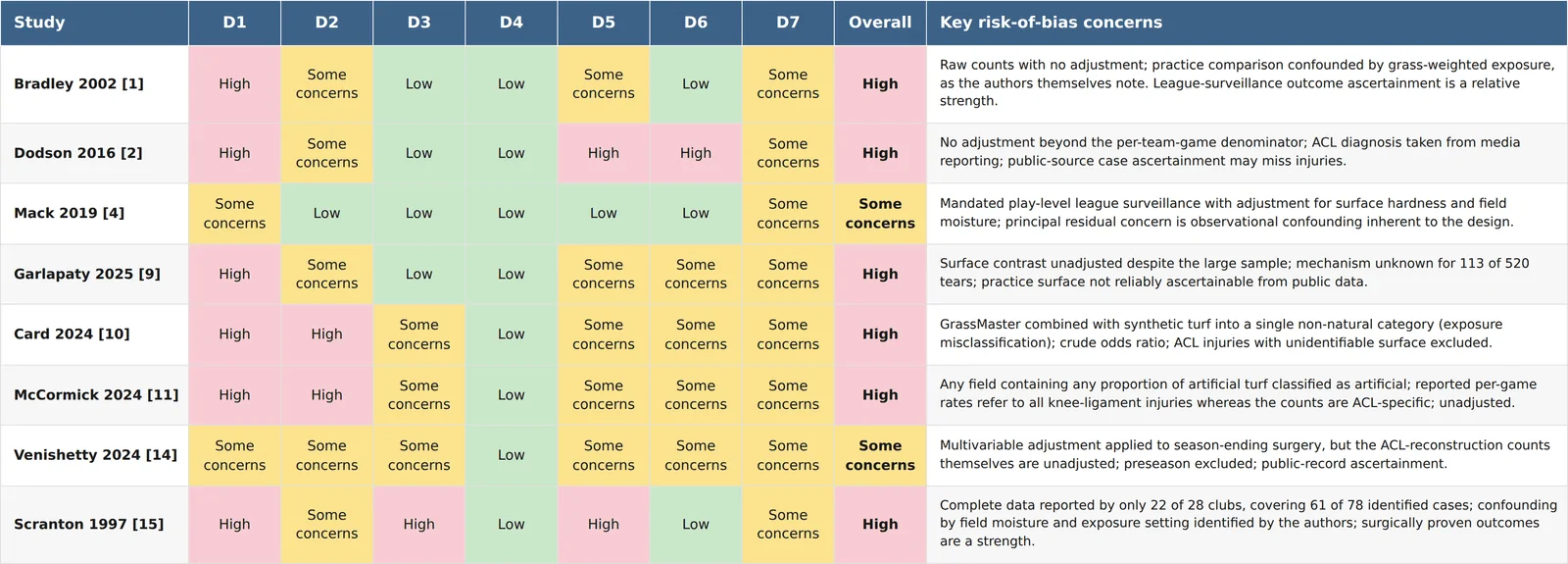
ROBINS-E risk-of-bias assessment ROBINS-E risk-of-bias assessment of the eight NFL observational studies. For each study, the result assessed was the comparison of ACL injury frequency or rate between artificial turf and natural grass; for Mack et al. [4] and Venishetty et al. [14], the closest surface-specific knee or season-ending surgery outcome. D1, confounding; D2, measurement of the exposure; D3, selection of participants; D4, post-exposure interventions; D5, missing data; D6, measurement of the outcome; D7, selection of the reported result. Judgements: low risk of bias/some concerns/high risk of bias/very high risk of bias. ROBINS-E: Risk Of Bias In Nonrandomized Studies-of Exposures; NFL: National Football League; ACL: anterior cruciate ligament

Risk-of-bias judgments are presented in Figure 4. Six of the eight NFL observational studies were rated at high risk of bias overall and two at some concerns; none reached low risk of bias, which is expected for observational exposure research of this type. Confounding stood out as the biggest concern: six studies looked at different playing surfaces but didn’t go further than basic comparisons like per-game or per-team-game rates. Only Mack et al. [4] and Venishetty et al. [14] took extra steps to account for other factors. When it came to exposure measurement, Card et al. [10] and McCormick et al. [11] were considered high risk because they labeled hybrid surfaces as artificial turf, which could skew the results. Notably, the two studies at the lowest risk of bias, Mack et al. [4], drawing on mandated play-level league surveillance, and Venishetty et al. [14], are also those reporting higher injury rates on synthetic turf. Using AMSTAR 2, we rated the included systematic review [5] as having low confidence, primarily because it did not specify which studies were excluded or explain the reasons for exclusion. We kept this review in the analysis for contextual purposes but did not use it as a source of incidence rates. The biomechanical study [3] does not present any data on the incidence of human injury. The main issue with this study is its generalizability, not its internal validity.

Discussion

This review synthesized evidence from NFL studies to assess whether artificial turf is associated with a higher rate of ACL injuries than natural grass. The central finding is that the apparent surface effect depends on how injury frequency is expressed.

Looking at raw counts, some NFL series have shown as many ACL injuries, or even more, on natural grass as on artificial turf [2,9,15]. When exposure is considered, however, the same datasets show comparable or higher rates on artificial turf. This held for the exposure-adjusted rates reported by Dodson et al., Garlapaty et al., and Scranton et al. [2,9,15]. Bradley found that ACL injuries occurred at similar rates on both surfaces during games. The higher number of injuries on grass was observed mainly during practices, suggesting that more practices are held on natural grass. McCormick observed a similar trend, with 81 ACL tears on turf and 50 on grass, though the overall rate of knee-ligament injuries did not differ markedly between surfaces [11]. When data from the four cohorts that tracked ACL injuries during games on both surfaces were combined, the analysis pointed to a rate ratio of 1.12 (95% CI: 0.89-1.40). Although the trend toward higher injury rates on artificial turf remained consistent even when each study was removed one at a time, the confidence interval was wide and included one. Since the cohorts covered overlapping years, these results should be viewed as suggestive rather than conclusive evidence of a difference between surfaces.

There are several reasons why a statistically significant difference in ACL injuries between surfaces is seldom seen in a single NFL study. ACL ruptures themselves are relatively uncommon events, and analyzing them by surface across just a few seasons often leaves studies with too little data to detect real differences, even when a true effect is present for all lower-extremity injuries. If injury rates are reported only as raw numbers without accounting for how often each surface is used, the comparison can be misleading. This is especially true since artificial turf is the setting for more regular-season games. In addition, the way outcomes are defined can affect how differences between surfaces appear. Differences between surfaces become more noticeable when studies include all lower-extremity injuries or focus only on noncontact or surface-contact injuries, which are most closely related to how the shoe interacts with the playing surface [3,4]. Another point to consider is that the surfaces studied span a wide time frame, from early AstroTurf in the 1980s to the latest infill systems. Combining results from these different eras can occasionally blur or dilute effects that are specific to modern surfaces.

When considered together, these different sources of evidence show a similar trend. NFL results adjusted for exposure suggest that ACL injuries are more likely on artificial turf. This pattern is supported by the pooled analysis, as well as by higher rates of knee injuries and noncontact injuries, which are most relevant to ACL tears, on synthetic surfaces [4]. More season-ending surgeries, especially ACL reconstructions, have occurred on turf [14]. Previous pooled analyses highlight American football as a sport where this increased risk stands out [5]. Laboratory findings at the cleat-surface interface provide further support for a mechanical explanation [3]. Although these results show that risk is higher on artificial turf, the NFL-specific ACL data alone do not conclusively prove this point. The results are also consistent with the wider football literature beyond the NFL cohorts synthesized here. Among college athletes, Loughran et al. observed a slightly higher rate of ACL injuries during competitions on artificial turf (rate ratio 1.18), though this difference was not statistically significant. They also found a significant increase in posterior cruciate ligament injuries [16]. In a similar vein, Dragoo et al. found that ACL injuries occurred more frequently on third-generation turf among National Collegiate Athletic Association (NCAA) football players, indicating the trend we observed [17]. Video analysis of professional ACL injuries also showed that most of these injuries occurred during noncontact movements like cutting and pivoting, which are likely related to the way shoes interact with the playing surface [18]. A recent scoping review reached a comparable conclusion, noting that the surface-ACL literature remains mixed and that higher-quality, exposure-adjusted data are needed [19]. Taken together with previous studies, our results suggest a consistent but small increase in ACL injuries on synthetic turf. While no single set of data can give a final answer, this trend shows up in different groups of football players.

These outcomes have immediate implications for both medical practice and league policy. Decisions about the type of field surface, how it is maintained, and even the design of cleats can all be adjusted to help limit injury risk. Many player advocates have pointed to injury data to support a return to natural grass. On the research side, future studies would be valuable if they consistently reported ACL incidence rates adjusted for exposure, distinguished between contact and noncontact injuries, and classified surfaces by specific generation rather than using a simple turf-versus-grass comparison.

Strengths and Limitations 

This review has several strengths. It focused specifically on NFL data, used clear study selection methods in line with PRISMA guidelines, and contained detailed extraction of surface-specific results from the source studies. The analysis also separated raw injury numbers from rates adjusted for exposure. There are, however, some important limitations. The search strategy relied solely on PubMed/MEDLINE, which was appropriate for the NFL focus of this review but might not have captured all relevant studies in other sources. This review was not registered in advance.

When reviewing the meta-analysis, it is also important to keep in mind that the four included cohorts span some of the same years. This coincidence means that some injury cases may have appeared in multiple studies. The studies do not have identical denominators, as some use games and others use team games. One study combined all non-natural surfaces into a single group for comparison with grass. Therefore, the pooled rate ratio should be viewed as descriptive rather than conclusive evidence. The body of evidence is also small, mostly retrospective, and sometimes relies on public data that could have errors. Additionally, two of the 10 studies, a multi-sport review and a biomechanical investigation, do not directly report NFL injury rates but provide a wider context for the findings.

In the future, research should prioritize collecting prospective, league-adjudicated data on ACL injuries that tracks ACL injuries by surface-specific athlete exposures. It would also help to report these injuries by contact mechanism and by the type of playing surface in use at the time. Consistent reporting across seasons would enable combining results and, eventually, producing a more definitive meta-analysis.

## Conclusions

In the NFL, most studies have not found a statistically significant increase in ACL injuries on artificial turf compared to natural grass. In some cases, the raw injury counts are higher on grass. After adjustment for how often each surface is played on, however, the rate of ACL injury is comparable or higher on turf. Pooling the game-level data in an exploratory analysis produced an estimate modestly above the null. This points toward a higher risk of ACL injury on turf, though the difference was not statistically significant and, given the overlap among the underlying cohorts, should be regarded as exploratory. Other sources of evidence add support to this trend. Other research has shown that knee injuries and noncontact injuries tend to occur more frequently on synthetic turf. More players undergo season-ending ACL injuries after playing on turf, and combined analyses across football highlight an increased risk, further supported by laboratory studies of surface mechanics. Although the NFL data alone do not provide a definitive answer, the overall trend in the evidence suggests a biologically plausible link between turf and higher ACL injury risk. More careful tracking of injuries, with attention to both player exposure and the details of how injuries happen, should make future research clearer. Until then, these results are relevant for player safety, field maintenance, and the league's standards.
